# The Role of eHealth in Optimizing Preventive Care in the Primary Care Setting

**DOI:** 10.2196/jmir.3817

**Published:** 2015-05-22

**Authors:** Mariko Carey, Natasha Noble, Elise Mansfield, Amy Waller, Frans Henskens, Rob Sanson-Fisher

**Affiliations:** ^1^Health Behaviour Research GroupPriority Research Centre for Health BehaviourUniversity of NewcastleCallaghanAustralia; ^2^School of Electrical Engineering & Computer ScienceUniversity of NewcastleCallaghanAustralia

**Keywords:** eHealth, Internet, prevention, general practice, family practice, evidence-based practice

## Abstract

Modifiable health risk behaviors such as smoking, overweight and obesity, risky alcohol consumption, physical inactivity, and poor nutrition contribute to a substantial proportion of the world’s morbidity and mortality burden. General practitioners (GPs) play a key role in identifying and managing modifiable health risk behaviors. However, these are often underdetected and undermanaged in the primary care setting. We describe the potential of eHealth to help patients and GPs to overcome some of the barriers to managing health risk behaviors. In particular, we discuss (1) the role of eHealth in facilitating routine collection of patient-reported data on lifestyle risk factors, and (2) the role of eHealth in improving clinical management of identified risk factors through provision of tailored feedback, point-of-care reminders, tailored educational materials, and referral to online self-management programs. Strategies to harness the capacity of the eHealth medium, including the use of dynamic features and tailoring to help end users engage with, understand, and apply information need to be considered and maximized. Finally, the potential challenges in implementing eHealth solutions in the primary care setting are discussed. In conclusion, there is significant potential for innovative eHealth solutions to make a contribution to improving preventive care in the primary care setting. However, attention to issues such as data security and designing eHealth interfaces that maximize engagement from end users will be important to moving this field forward.

## Importance of Preventive Care in Optimizing Health Outcomes

### Background

Modifiable lifestyle risk factors such as being overweight, smoking, poor nutrition, excess alcohol consumption, and physical inactivity are among the major causes of morbidity and mortality worldwide [1,2]. These risk factors contribute significantly to the development of chronic diseases [3], which are the leading causes of death globally [4]. In 2002, chronic diseases including cardiovascular disease, cancer, chronic respiratory disease, and diabetes caused 29 million deaths worldwide [5]. The estimated annual economic impact of chronic diseases including cancer, heart disease, and diabetes in the United States in 2007 was US $1.3 trillion, including US $277 billion in direct treatment costs [6].

### Role of General Practice in Delivery of Preventive Care

Implementation of best practice preventive care has the potential to substantially improve health outcomes by reducing the prevalence of modifiable risk factors. Primary health care is well positioned to address the challenges of chronic disease prevention and management [7], with each health care visit being a potential opportunity to provide preventive care [8].

Prevention is recognized by both general practitioners (GPs) and patients as one of the key roles of GPs [9,10]. The effectiveness of brief interventions (defined as short, motivational, patient-centered interactions) by GPs in encouraging changes in weight, alcohol, smoking, and physical activity behaviors has been demonstrated [11-13].

### Improvements in Preventive Care Urgently Needed

Despite the development of national guidelines and acknowledgement by GPs of their professional responsibility in management of lifestyle risk factors [14], actual rates of preventive risk factor screening and management remain low [15,16]. For example, GPs rarely engage in lifestyle counseling with obese patients during their regular consultations [17]. Further, while many GPs report using verbal counseling for risk factors such as lack of physical activity, they rarely provide referrals or written action plans [18]. The gap between recommended care and actual delivery rates is further underscored by patient reports of a desire for more lifestyle advice [16]. Such findings indicate that there may be barriers affecting GPs’ ability to screen for and provide advice on risk factors.

### Barriers to Best Practice Preventive Care in Primary Care

Barriers to best practice preventive care include a lack of practitioner time, skills and reimbursement, and low patient motivation [19-21]. A recent review showed that practitioner time was the most frequently cited barrier to detection of lifestyle risk factors [22]. Preventive care must be balanced with already limited time available to deal with both immediate and ongoing health conditions. One US study estimated that in order to provide all the preventive services recommended by the US Preventive Services Task Force, each physician would be required to spend 7.4 hours per working day on prevention alone, highlighting the difficulties in meeting current preventive care recommendations [23]. Strategies for delivering time-efficient yet comprehensive lifestyle risk factor management in primary care are therefore required.

### The Promise of eHealth in Improving Preventive Care

The disparity between recommended preventive care and actual screening behavior has prompted a call for alternative methods for collecting patient health information. eHealth technologies represent one strategy for improving the accuracy and completeness of clinical information collected from patients. eHealth is the “intersection of medical informatics, public health, and business, referring to health services and information delivered or enhanced through the Internet and related technologies” [24]. The use of information and communication technologies to improve health is rapidly expanding. These technologies can be used to gather, manage, and disseminate health information via computers, tablets, and mobile devices [25]. Electronic data collection via these portable devices offers a number of significant advantages for the assessment and management of patient lifestyle risk factors. eHealth technologies can support clinical practice by facilitating the accessibility of patient data and appropriate evidence-based guidelines, offering a potential strategy for improving the safety, quality, and efficiency of care [26,27].

## Improving the Comprehensiveness and Accuracy of Clinical Information

### Assessments of Lifestyle Risk Factors

Electronic assessment of lifestyle risk factors can be implemented prior to a patient’s consultation with their GP, so that the information can be transmitted instantaneously to the GP and addressed during routine encounters. These assessments therefore provide valuable real-time clinical information that can help guide the consultation and facilitate opportunistic intervention. Multiple risk factors can be assessed simultaneously to ensure that a comprehensive picture of the patient’s situation is available.

### Acceptability to Users

Several studies have demonstrated the acceptability of electronic health assessments administered in waiting rooms in general practice clinics. Our study of over 4000 patients from 12 Australian general practices found that 86% of those eligible were willing to complete an electronic health risk assessment on a touchscreen computer in the waiting room [28]. The vast majority of patients reported that the system was easy to use (94%), and 77% of patients were willing to have GPs keep their survey responses on file [28]. Similar findings have been reported in studies from New Zealand, the United Kingdom, and the United States [29,30]. Patients report that electronic assessments are sufficiently private (91%) [30] and indicate a preference for electronic approaches over paper and pen assessments. Support for the implementation of repeated assessment is also available, with 86% of patients and 90% of GPs indicating tht they would be willing to complete electronic assessments at future consultations [31].

### Feasibility and Acceptability to Clinic

GPs have expressed concerns in relation to the integration of patient risk factor assessments into routine practice, perceiving potential burden on staff and disruptions to the clinic, such as increased waiting times and consultation length. However, our data showed 89% of patients were able to complete a comprehensive health risk survey in less than 15 minutes, and 99% were able to do so prior to their consultation [28]. Given that the majority of general practice patients wait on average 11-30 minutes before an appointment [32], the completion of electronic assessments prior to consultation is highly feasible. Our data indicate that this approach does not disrupt the clinic, increase patient waiting times, or increase staff burden [28]. As many patients consult their GP several times a year (on average in Australia, 6.5 times per year [33]), implementation of this approach enables tracking of health risk factors over time.

### Accuracy of Self-Reported Data

Clinicians primarily rely on patient self-reported risk factors when assessing a patient’s medical history. While more accurate assessments such as cotinine analysis for smoking [34] or blood alcohol tests may be used [35], these are generally too intrusive, expensive, and time consuming to be used for routine screening of health risk factors. Although the accuracy of self-report data may be affected by factors such as social desirability and recall biases, for many lifestyle risk factors, self-report is the most feasible method of assessment [34,35]. Inconsistencies in questions used by clinicians, however, can result in variable accuracy of self-reported health behaviors [36]. The use of an electronic health risk assessment may help overcome this by allowing standardized questions, with established reliability and accuracy, to be used across all patients.

### Simplification of Complex Assessments

The assessment of some lifestyle behaviors can be complex. For example, quantity and frequency assessments of alcohol intake require the respondent to not only recall the frequency of intake, but also to accurately assess what volume of different types of alcohol constitutes a standard drink [35]. Some of these complexities can be overcome in electronic risk assessment by using dynamic elements to simplify assessment. For example, electronic assessment tools for alcohol may allow participants to select the type and number of drinks they have consumed, with the program automatically converting these into standard drinks [35]. These types of strategies have been used in electronic surveys to help improve accuracy of reporting [35].

### More Comprehensive Assessment of Risks

As noted above, GPs often have limited time for preventive care during a consultation and therefore may screen for only a limited range of risk factors, if at all. In contrast, electronic health assessments completed prior to a consultation can efficiently cover a standardized and comprehensive range of risk factors. Branching algorithms can be used to tailor the assessment and ensure participants are not required to answer irrelevant questions, thereby minimizing required assessment time. This information can then be automatically summarized and fed back to GPs prior to the patient’s consultation, with areas that require risk management flagged.

## Improving Provision of Clinical Care, Including Self-Management Advice

### Point-of-Care Feedback

Computing systems have the capacity to use collected information to design personalized health programs or provide point-of-care individualized feedback [37]. If appropriate risk behavior information is collected, point-of-care feedback on patient risk factors can be provided to both the patient and clinician in real time, either as an onscreen display or in print [37,38]. Such feedback can be used as a reminder to prompt discussion of preventive care issues within the consultation. One review found that computerized feedback produced modest changes in clinical behavior [38]. However, it is notable that the review focused on a range of clinical behaviors, with only a few preventive care activities included. This suggests that there is a need to further investigate the impact of computerized feedback on a broader range of preventive care practices. This process can assist in streamlining consultations, increasing the time available for the delivery of advice or referral to other services or specialist providers. If consultation time is particularly limited or other urgent health care issues need to be addressed, there is potential for patient feedback to be uploaded to the patient’s electronic medical record for discussion at a subsequent appointment.

### Focused Secondary Screening by General Practitioner

By providing GPs and patients with the results of the electronic assessment, GPs can quickly identify which health issues are of concern and provide a more in-depth assessment, such as exploring the severity and impact of the health risk, as well as the social, psychological, medical, and environmental context that contributes to or exacerbates the risk factor. Through reducing the time burden associated with risk assessment and summarizing existing risk behaviors, electronic screening and feedback maximizes the time available for the provision of preventive care, thus allowing GPs to use their consultation time more effectively.

### Results Available to Multiple Health Providers

If the GP refers their patient to specialist or other follow-up care, electronic screening results can also be made available to the other relevant providers. This reduces the need for replication of risk assessment by additional providers, again allowing other health care providers to use their time with the patient more effectively. There is some evidence that electronic sharing of medical information among clinicians within and across settings improves continuity of care [39].

### Promoting Patient-Centered Care

Patient-centered care is concerned with ensuring that care provided is in accordance with patients’ needs, values, and preferences [40]. Given that changing lifestyle behaviors require active and ongoing partnership from the patient, it is particularly important that preventive care takes a patient-centered approach that incorporates the needs and goals of the person [41]. Interventions that are matched to a participant’s stage of change have shown promise for improving some behaviors [42,43]. It follows that adherence is likely to be greater if the recommendations are congruent with patient values and motivations. However, clinicians also have limited time to identify patients’ preferences and needs in order to tailor their care. Electronic health assessments can help overcome this by including a systematic assessment of patients’ priorities or readiness to change with regard to lifestyle risk factors. In situations with no clear clinical reason for prioritizing change of one lifestyle risk factor over another, this information is likely to be useful in guiding clinicians to target discussion or advice towards patient priorities or readiness to change.

### Recall and Reminder Systems for Patients

Recall and reminder systems involve an automated system to trigger a reminder to the patient to perform a routine action or test. These systems may trigger a letter, telephone call, short message service (SMS), or email prompt. Recall and reminders have been used successfully to help patients manage chronic and complex diseases such as diabetes [44,45]. Although applications to preventive care have been less widespread, reviews suggest that recall and reminder systems are also likely to be effective in preventive care [46,47]. A Cochrane review found that recall and reminder systems were effective in improving immunization rates among both adults and children [46]. Multiple reminders were more effective than single reminders, and telephone reminders were more effective than mailed reminders [30]. Reminder systems for breast and colorectal screening have also been shown to improve patient uptake of such tests [47]. Potential applications of recall and reminder systems to prevention of lifestyle risk behaviors include providing automated reminders to clinicians to follow up on advice provided in previous consultations and prompting the provision of additional tips or suggestions that encourage the patient to adhere to treatment plans.

### Reminder Systems for Clinicians

Computerized reminder systems for clinicians may involve reminders delivered electronically (eg, an alert on the computer screen) or via paper. Point-of-care reminders have been shown to be effective in prompting health care providers to perform a patient- or encounter-specific clinical action [48] and in improving physician adherence to processes of care [38]. Computerized reminders delivered on paper have been shown to improve care by a median of 7% [49]. Reminders that provided space for the clinician to record a response or explanation were more effective than those without this feature [49]. While studies to date have demonstrated that this type of intervention can be effective for increasing some preventive care behaviors such as participation in screening for cancer [49], there is a need for examination of how this can be applied to other types of preventive care such as addressing lifestyle risk factors. In the context of a broader range of preventive care, clinician reminder systems could be used to remind clinicians to monitor progress with lifestyle changes, reassess risk factors, or to administer a test or specific clinical action.

### Provision of Tailored Educational Materials and Web-Based Resources

Self-management is the frontline intervention for most lifestyle risk factors. Even when risk factor severity indicates the need for pharmacological intervention, self-management is still required to ensure adherence to a recommended medication regime. The ability to initiate and sustain risk factor change depends on several factors, including patient awareness of the harm caused to health by particular behaviors, and the desire and ability to change [50]. Lack of knowledge about disease and treatment is one of the major obstacles to compliance with treatment [51]. There is also evidence to indicate that patients are often ill informed about their risk factors and how to manage them. For example, Silagy et al reported low awareness of the risks associated with a high fat diet [52]. Similarly, only one out of three established cancer risk factors for five common cancers were identified by British adults [53], and the majority of Americans were unable to identify major risk factors for breast, cervical, and colon cancers [54]. Therefore, patients may need information about their risk factors as well as the changes they should make to reduce these risk factors.

Electronic risk assessments may be programmed so that they either (1) generate tailored information on self-management of risk factors that can be printed in clinic, or (2) refer patients to specific online eHealth programs that provide advice and interactive self-management tools to help manage risk factors. The latter can be done by sending links to relevant programs to the patient’s email address.

There is emerging evidence for the effectiveness of eHealth interventions for a range of health behaviors. For example, systematic reviews have found that interactive computer-based interventions are effective in producing small reductions in weight among overweight and obese people [55] and increasing knowledge about sexual health among various populations [56]. However, more evidence is needed, particularly evidence specific to the primary care setting. A recent review identified that no studies had evaluated the impact of Web-delivered physical activity interventions over a 12-month period or longer, and none in general practice settings [57]. Similarly, another review found only mixed evidence for the impact of Web-based interventions for smoking cessation; however, none of these studies were specific to the primary care setting [58]. This suggests that there is considerable scope to develop and test eHealth interventions for primary care populations.

### Potential Advantages of Web-Based Self-Management Resources

#### Flexibility of Presentation

Web-based materials can be presented in a variety and combination of formats including text-based, verbal (eg, audio or embedded videos), and visual (eg, graphs, pictures, or animations) information. Provision of information in multiple formats improves comprehension, particularly for less literate patients [59].

#### Enhanced Relevance to the Reader

Web-based programs can be interactive such that the user can input details about their health concerns or health status and be directed to tailored information. In addition to saving the user time in searching through irrelevant information, there is strong evidence that tailoring improves recall and comprehension of medical information [59]. Studies also indicate that there is variation among individual patients in the level of detail that they prefer [60] and that tailoring to such preferences reduces patient anxiety [61].

#### Standardization of Care

There is considerable variation with respect to many aspects of health care delivery, including within primary care. This is in part due to the time pressures of clinical practice, differences with respect to services and systems within health care organizations, and differences in the skills and knowledge of clinicians [62]. There is also evidence that patients residing in rural areas, for example, experience more difficulties in accessing face-to-face services due to limited availability of primary care services [63]. Online approaches can ensure that high-quality, evidence-based information is made available to all patients, overcoming potential inconsistencies including those due to geographical barriers.

#### Adopting Evidence Into Practice

eHealth applications provide a central mechanism for disseminating and maintaining evidence-based information with broad population reach. Information can be centrally updated to correspond with changes in guidelines to ensure that the latest and best-evidence practices are disseminated to patients.

#### Accessibility in a Range of Languages

Interactive Web-based programs can be programmed so that the user can select the language the material is presented in on screen. This has advantages for multicultural countries such as Australia, the United Kingdom, and United States and can help ensure that people who are not fluent in English are not disadvantaged.

#### Enhanced Recall and Understanding

Strategies used for written materials to enhance comprehension and recall can also be applied online. These include explicit categorization [64,65], use of plain language [66], and repetition of important pieces of information [67].

#### Linkage to Data Provided in Other Websites

Web-based programs can be configured to collect and display information from other credible websites. This could include, for example, presentation of up-to-date health statistics, clinical information, and research and policy information.

## Challenges of Using eHealth in Primary Care

###  Will eHealth Exacerbate Disparities in Care?

According to the World Bank, Internet access continues to rise globally. In 2011-2013, high rates of Internet access were reported for developed countries such as the United States (84%), Australia (83%), Germany (84%), Japan (86%), and the United Kingdom (90%) [68]. However, there is a risk that eHealth applications will exacerbate health disparities among groups with lower Internet access and/or skills. One potential risk is that particular patients will be unwilling or unable to use computer-based health assessments administered in clinic. Our pioneering work in the late 1990s, however, indicated the acceptability of touchscreen computers in a variety of community and specialist health care settings including general practice [69], drug and alcohol clinics [70], and oncology settings [71]. Since then, the mainstream use of touchscreen technology on computer tablets and mobile phones has increased considerably. Our recent multisite study of general practice care indicated that more than 90% of patients rated the touchscreen health assessment administered in the waiting room as highly acceptable [28]. High rates of acceptability ranging from 88% to over 90% have also been confirmed in community settings serving socioeconomically disadvantaged clients [72,73]. This suggests that with appropriate survey tools, socioeconomic factors will not necessarily be a barrier to the implementation of this technology as part of standard clinic care.

There are perhaps greater risks of disparities where patients are referred to use eHealth applications outside the clinic. These relate to both disparities in access to the Internet, and in engagement with, and ability to apply, the information provided, in order to improve health. Several studies have reported differences in Internet access among subgroups of the population such as older people [74,75], racial minorities [76], and people who are socioeconomically disadvantaged [75,76]. However, there is evidence to suggest that the “digital divide” is becoming narrower as more people gain access to the Internet [77].

In relation to engagement and use of information, developers of eHealth programs can potentially incorporate design features to overcome such barriers. As described earlier, there are many features that can be built into the design of eHealth programs such as the use of language, layout, and graphics that can mitigate poor health literacy. As the digital divide narrows, the issue of how to ensure that information can be understood and applied by a wide range of people is likely to become increasingly prominent [77,78]. A client-centered approach that maximizes the user’s experience of interacting with the technology and enhances its ease of use is needed. This involves iterative development that incorporates user feedback [79]. Human factors research advocates a range of factors that need to be taken into account when designing eHealth applications including readability and ease of navigation of the interface, user skills training needs, and how easily and efficiently the interface allows the user to complete necessary tasks [80].

### Integrating Patient Electronic Assessments Into Existing Practice Software Systems

Data obtained using electronic assessment tools can be initially collected and stored by the Web server software executing on the server that provides the webpages. The data are aggregated on a per-page basis during communication between the server and the device (eg, tablet computer) on which the patient performs their assessment. Ideally, the collected assessment data would then be made available on each respective patient’s medical record. This copying of patient data between software systems is similar to the current widely implemented transfer of pathology, radiology, and other data/images from laboratories and collection centers to GP practice software.

Implementation of the transfer of data is typically achieved by transfer of messages between the data producer (in this case the Web-based assessment system) and the practice software. As envisaged by McDonald et al, both parties to the data transfer need to “understand” an agreed upon message format used by the Web-based assessment system to send the assessment data to the practice [81]. The ANSI-accredited Health Level 7 standards development organization (HL7) [82] aims to standardize interoperability, so that transfer between medical software systems is straightforward. For example, the practice software may “understand” the HL7 compliant Medical-Objects [83] format for medical message transfer. While most current medical practice software would support HL7 communication, if the practice software does not support such message transfer, the Web-based assessment system could automatically send each patient’s assessment (in a format such as csv) to a provided email address (representing the practice) for manual import into the practice software. It is expected that manual import would, however, be required only as an interim solution for the minority of practices using out-of-date, non-connected software.

### Ensure Use of Electronic Assessment Results by General Practitioners

Simply providing risk factor results to GPs may not ensure that results are utilized by GPs (although as noted above, the use of point-of-care reminders have been shown to be effective in improving care). For example, Brindle et al found no strong evidence that cardiovascular risk assessment performed by a clinician improved patient health outcomes, possibly due to the poor uptake of computerized clinical decision support systems [84]. Therefore, electronic risk factor assessment results need to be seen as relevant and useful by GPs. Including clinicians in the design of the assessment or results may improve their use [84]. Providing links to relevant guidelines, and/or advice for GPs about recommended actions or next steps, may also help ensure the clinical utility of electronic risk assessment results.

### Ensuring Security of Patient Data

Security of data is three-fold: (1) the device on which the data are collected must prevent unauthorized users from accessing data or immediately transfer the data to another site so it is not locally stored, (2) any devices used to store data must control physical access by implementing, for example, password-based access control, and be protected against unauthorized external access using mechanisms such as firewalls controlling Internet traffic, and (3) data must be rendered unreadable using encryption techniques that allow decoding by the correct recipient and prevent decoding by unauthorized interceptors of the data. Each of these methods of securing data is currently available. For example, an electronic risk assessment on a portable device (such as tablet computer) could use a local Web browser to receive webpage content from the Web server and send patient responses back to the Web server at the completion of each page of the assessment (signaled when the patient clicks the “next” button on their screen). In this way, no patient data need be stored on the tablet between assessments, and access by unauthorized persons would be impossible on the data collection device. A patient’s response to each page of the risk assessment would be encrypted while in transit between the tablet computer and the Web server, thus preventing its being understood in the event of interception. The centralized server would be positioned within a secure data center with appropriate access control preventing unauthorized internal and/or external access to patient data. Such methods could be used to ensure security of patient data within the electronic risk assessment approach proposed in this paper.

### Conclusion

There is great potential for eHealth to assist clinicians in assessing preventive health care needs and in enhancing the delivery of care to manage such risks. While there are practical challenges that need to be considered in the implementation of eHealth programs, these are not insurmountable. Engagement of end users (patients and clinicians) in the development of such applications, and ensuring data security concerns are addressed will be crucial to advancing this field.

## Abbreviations

GP: general practitioners
SMS: short message service
